# Demonstrating the learning and impact of embedding participant involvement in a pandemic research study: the experience of the SARS-CoV-2 immunity & reinfection evaluation (SIREN) study UK, 2020–2023

**DOI:** 10.1186/s40900-023-00506-6

**Published:** 2023-10-18

**Authors:** Anna Howells, Erika Neves Aquino, Deepika Bose, Martin Gerard Kelly, Barbara Molony-Oates, Asmah Hassan Syed, Kim Tolley, Claire Neill, Susan Hopkins, Victoria Hall, Jasmin Islam

**Affiliations:** 1 UK Health Security Agency, Nobel House, London, SW1P 3HX UK; 2 British Society for Immunology, 9 Appold Street, London, EC2A 2AP UK; 3 Medical Offices, Stratheden Hospital, Cupar, KY15 5RR UK; 4 Altnagelvin Area Hospital, Glenshane Road, Derry, BT47 6SB UK; 5 Health Research Authority, 2 Redman Place, London, E20 1JQ UK; 6 Lister Hospital, Stevenage, SG1 4AB UK; 7 The Health and Care Professions Council, Park House, 184-186 Kennington Park Road, London, SE11 4BU UK

**Keywords:** COVID-19, Pandemic response, PPI, Patient and public involvement, Participant involvement, Participant engagement, SIREN, SARS-CoV-2, Coronavirus

## Abstract

**Background:**

Participant involvement in research studies is not a new concept, yet barriers to implementation remain and application varies. This is particularly true for pandemic response research studies, where timeframes are condensed, pressure is high and the value and inclusion of participant involvement can be overlooked. The SIREN Participant Involvement Panel (PIP) provides a case study for participant involvement in pandemic research, working in partnership with people who the research is for and about.

**Methods:**

SIREN and the British Society for Immunology (BSI) recruited and ran two phases of the PIP, involving 15 members in total over a 16-month period. Phase 1 ran between January and August 2022 and Phase 2 between October 2022 and March 2023. Activity figures including recruitment interest and PIP meeting attendance were recorded. To evaluate how the PIP has influenced SIREN, feedback was collected from (a) researchers presenting at the PIP and (b) PIP members themselves. Evaluation at the end of Phase 1 informed our approach to Phase 2. Thematic grouping was planned to identify key lessons learned.

**Results:**

Applications increased from n = 30 to n = 485 between Phase 1 and Phase 2 of the PIP, a more than 15-fold increase. The SIREN PIP positively impacted the design, implementation and evaluation phases of the study and sub-studies. Feedback from PIP members themselves was positive, with members highlighting that they found the role rewarding and felt valued.

Learnings from the PIP have been condensed into five key themes for applying to future pandemic response research studies: the importance of dedicated resources; recruiting the right panel; understanding motivations for participant involvement; providing flexible options for involvement and enabling the early involvement of participants.

**Conclusions:**

The SIREN PIP has demonstrated the value of actively involving people who research is for and about. The PIP has provided an active feedback mechanism for research and demonstrated a positive influence on both SIREN study researchers and PIP members themselves. This paper makes the case for participant involvement in future pandemic research studies. Future work should include improved training for researchers and we would support the development of a national PIP forum as part of future pandemic research preparedness.

**Supplementary Information:**

The online version contains supplementary material available at 10.1186/s40900-023-00506-6.

## Abstract (plain language summary)

The SARS-Cov2 Immunity & Reinfection Evaluation (SIREN) study was set-up at speed during the early stages of the pandemic to help answer key questions about COVID-19 and inform the national pandemic response. It has provided valuable insight into COVID-19 infections, reinfections, and how well the vaccines work. SIREN helped to find these answers by regularly testing over 44,000 healthcare staff working at 135 NHS organisations. To support participant retention, SIREN established a Participant Involvement Panel (PIP) involving 15 SIREN participants to date. PIP members provide guidance and feedback to SIREN researchers on key research priorities, changes to the study and strategies for maximising participant engagement. This paper provides insight into how the PIP was set-up, run and the resources required from the perspective of the PIP and SIREN researchers. Lessons learned from establishing the PIP are summarised to help inform future pandemic response research studies. The paper adds to the evidence base, and makes the case for, the valuable role participant involvement can play in pandemic response research studies.

**Supplementary Information:**

The online version contains supplementary material available at 10.1186/s40900-023-00506-6.

## Background

### What is the SIREN study?

The SARS-CoV-2 Immunity and Reinfection Evaluation (SIREN) Study is a prospective multicentre cohort study established to evaluate the immune response to SARS-CoV-2 amongst healthcare workers [1]. SIREN enrolled over 44,000 participants between 18 June 2020 and 31 March 2021. Study participants are NHS staff working at healthcare organisations that have joined the study as SIREN sites located across the four UK nations. A participant was eligible to join the study if they worked in a clinical setting where patients are present, which included administrative, executive and support staff. SIREN participants undergo fortnightly PCR tests, monthly or quarterly serology and regular questionnaires. Follow-up was for an initial 12 months, with the option to extend follow-up for a further 2 years.

The SIREN study has been instrumental in answering questions regarding immunity to SARS-CoV-2 infection, informing national vaccination strategies and functioning as a surveillance tool for emerging SARS-CoV-2 Variants of Concern [2–5]. The SIREN study is a multidisciplinary collaboration of public health policy makers and academic research partners. The British Society for Immunology (BSI) have worked collaboratively with the UK Health Security Agency (UKHSA) SIREN study team to lead the recruitment, organisation, facilitation and evaluation of the SIREN PIP.

#### Defining participant involvement & establishing the PIP

There is growing consensus that involvement from the people who research is for and about should form an integral part of research studies [6–10]. Benefits of actively involving service users (study participants, patients, the public or otherwise) include generating higher quality and more relevant research owing to “the unique perspective that users can bring to a research project” [6]. There is also a moral argument, that those affected by research “should have a say in what and how it is done” [7], and that participant involvement “can result in a higher societal benefit through better use of resources for research” [8]. Participant involvement can achieve this through the identification and selection of high-priority research questions, planning and performing of more focused research, and improving participant enrollment [8]. In the context of longitudinal cohort studies specifically, a recent qualitative study highlighted that participant input can “improve study designs, make them more acceptable for uptake by participants and aid in contextualising research communication to participants” [9]. These elements become increasingly important as the length of study increases, as is the case with the SIREN study.

SIREN was established early in the COVID-19 pandemic response, with recruitment scaled up at pace due to the nature of the pandemic. SIREN acknowledged that involvement from NHS staff participating in the study would be key to participant retention, and that input from participants could provide valuable feedback to help guide and improve the study.

Research studies referencing stakeholder involvement work commonly refer to Patient and Public Involvement (PPI) or Public Involvement. The former can be defined as “an active partnership between patients and the public and researchers in the research process, rather than the use of people as ‘subjects’ of research” [11] and the latter defined as “patients or other people with relevant experience contribute to how research is designed, conducted and disseminated” [12]. SIREN has adopted and adapted these principles. To work in partnership with the people who the research is for and about, SIREN has created an active partnership with study participants through the PIP. This is appropriate because the study focuses on the health of healthcare workers with results applicable to this population group.

The SIREN PIP are a group of SIREN study participants who meet on a regular basis. The aims of the SIREN PIP are to provide open, honest feedback and guidance to SIREN researchers on (a) research priorities and study protocol changes, including emerging sub-studies, (b) operational elements of study delivery, including cohort retention, and (c) the wider implications of SIREN research.

### Aims

This paper aims to outline our approach to participant involvement in a large, multicentre pandemic response cohort study, providing a narrative account co-produced by PIP members and researchers. We aim to demonstrate the impact and value of participant involvement in the SIREN study and identify learnings for future studies, following the GRIPP2 [13] framework for reporting patient and public involvement in research where possible (Additional File 1).

### Contribution to field

With a reduction in public involvement in research documented during the COVID-19 pandemic [14] it is important to highlight examples of where this did take place, to demonstrate its impact and prevent this happening in future. Our paper provides an example of participant involvement embedded within a unique, large-scale prospective cohort study established during the COVID-19 pandemic.

## Methods

### Stages of involvement

Phase 1 SIREN PIP began in January 2022 and ran until August 2022. Phase 2 of the PIP began in October 2022 and ran until March 2023.

*“The PIP was transformative in how we approached cohort retention activities – enabling us to expand from participant engagement to include participant involvement.”*

- *Researcher 1, UKHSA SIREN Team*

### PIP recruitment

Recruitment for Phase 1 was announced in a study-wide participant newsletter. Phase 2 recruitment methods were expanded through using a live webinar, recurrent newsletter advertisements and by utilising individual SIREN site internal communication pathways for participants. Information about the PIP was hosted on the BSI website and included an overview of its aims, responsibilities of members, ways of working, a code of conduct, honorarium details and a link to an online form for participants to express an interest in joining the panel. The form collected contact and demographic details with patient consent.

*“It seemed simple to do…I felt like I was giving something back”*

- *Participant 1, SIREN PIP member*

**Fig. 1:**
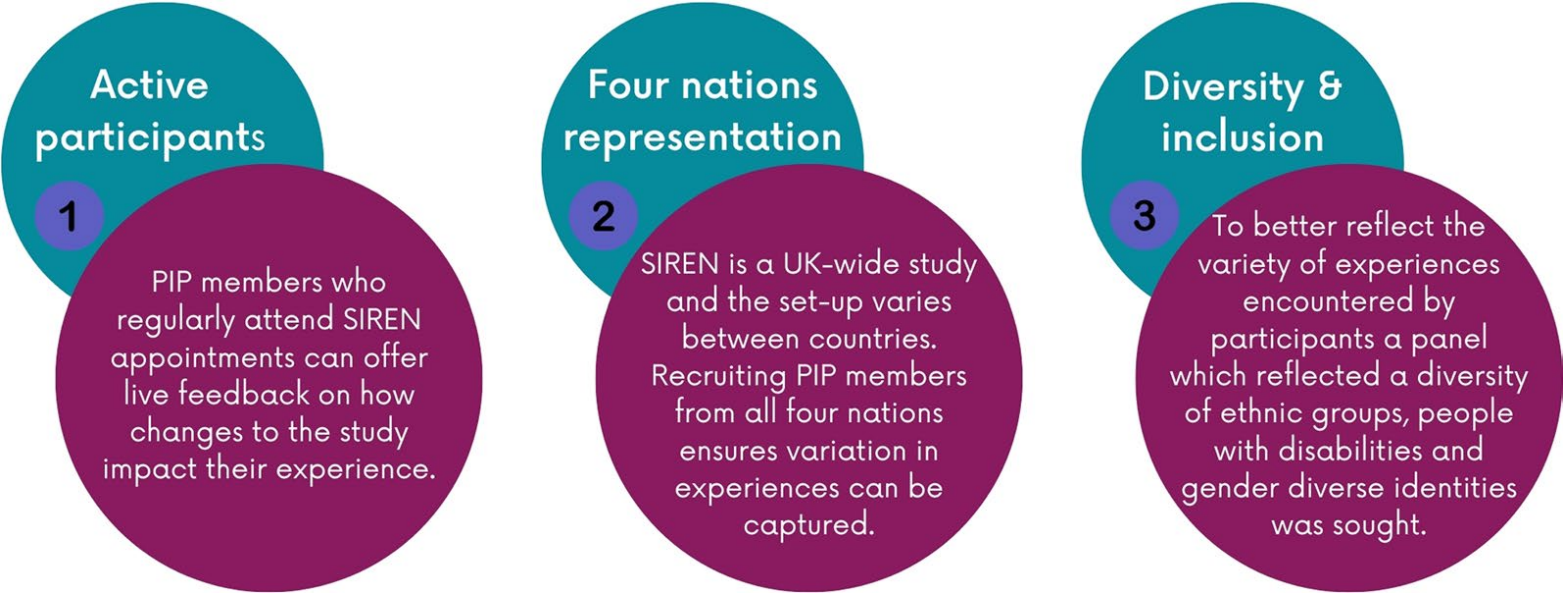
The criteria used to select PIP members in Phase 2

PIP expressions of interest were reviewed by the BSI and a shortlist of candidates was produced. We sought to recruit a panel that met three criteria for Phase 2. (Fig. 1). Expressions of interest from participants meeting these criteria were encouraged through an explicit statement on the recruitment webpage.

*“Diverse group of people, fab to see people from across the UK and from different roles.”*

- *SIREN PIP member, Phase 1 evaluation*

The BSI held informal conversations with shortlisted individuals over Zoom [15], to understand their motivations for joining, ensure individuals could commit to the time requirements involved and to confirm they would be willing to provide open and honest feedback on the topics discussed in meetings.

### PIP members

Seven study participants were recruited for Phase 1 of the PIP and 10 for Phase 2. Two members of the Phase 1 panel continued into Phase 2, based on their interest to remain and active study status. In both phases SIREN PIP members represented a range of professional groups including frontline staff (clinicians, midwives, nurses), those working in administrative or executive roles, and staff working in estates, porters or security staff groups.

### Running the PIP

Meetings were held on a six-weekly basis and took place via Zoom to enable whole-group discussion. Each meeting was co-chaired by the BSI and a volunteer PIP member co-chair. The length of meeting varied from 1.5 to 2 hours.

PIP meetings aimed to provide an inclusive environment, encouraging everyone to share their experiences and perspectives; in line with the original intention of encouraging active participation. Panel members were asked to abide by the BSI code of conduct.

*“With trepidation, I attended the first meeting. Immediately, there was great warmth & inclusiveness. I felt that my thoughts & opinions did matter.”*

- *Participant 1, SIREN PIP member*

Availability for meetings was assessed via Doodle poll [16] and the date and time chosen was based on highest availability, with the option of evening meetings available in Phase 2.

*“The periodic meetings held for PIP members kept us updated about the progress of research, gave us the opportunities to address our queries, reflect on the challenges, and make suggestions to incorporate in the research process. This gave a feeling of ownership of the research and made me want to remain a dedicated volunteer.”*

- *Participant 2, SIREN PIP member*

Members were contacted in advance of each meeting with an agenda and briefing documents. Meeting agendas were coproduced by BSI and a UKHSA SIREN team member. Meeting topics were based on expressions of interest from SIREN researchers and academic partners. Feedback from PIP members also informed meeting topics, with a focus on inviting presenters to return and share their feedback on how the PIP helped shape their work.

At the end of each meeting PIP members were offered an honorarium of £50, in line with the National Institute for Health and Care Research (NIHR) payment guidance [17]. Minutes and agreed actions were shared following each meeting.

*“The meetings were well organised with plenty of time for discussions, papers always sent out in advance, and a comprehensive set of minutes were circulated afterwards.”*

- *Participant 3, SIREN PIP member*

**Fig. 2:**
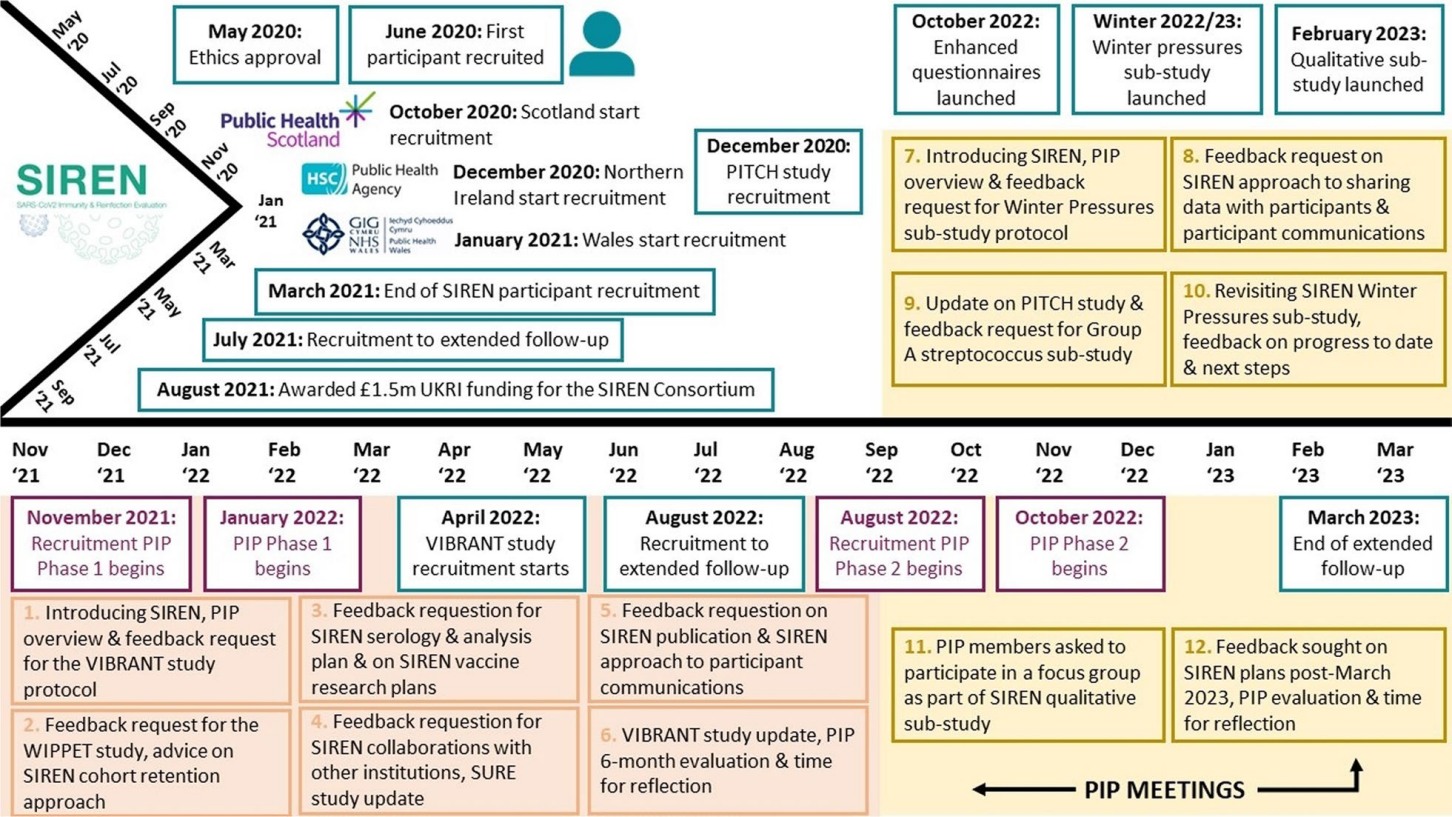
A timeline of key SIREN study moments in addition to PIP meeting activity and topics from May 2020 to March 2023. Phase 1 PIP meetings are highlighted in orange with Phase 2 PIP meetings highlighted in yellow

A timeline of SIREN study and PIP activity can be found in Fig. 2.

### Measuring the influence of the PIP

Feedback was captured to understand the influence of the PIP. Feedback was collected from PIP members and presenting researchers at the end of each meeting, and from PIP members at the end of each phase. Methods to obtain feedback included online surveys featuring open text responses and Likert scales, and designated feedback tools such as Padlet [18].

*“Anonymous feedback via the Padlet app was excellent & led to improvements in the meetings themselves.”*

- *Participant 1, SIREN PIP member*

## Results

### PIP activity

Two phases of the SIREN PIP took place between January 2022 and March 2023. Interest in applying for the SIREN PIP increased throughout the study from 30 participants for Phase 1 to 485 for Phase 2, a more than 15-fold increase in applications.

*“We announced Phase 2 recruitment during a celebratory webinar for participants which included a PIP member speaker. We highlighted the PIP in newsletters and set aside over a month for participants to apply, as we were conscious that it was the summer period when many staff take time off.”*

- *Researcher 2, UKHSA SIREN team*

11 SIREN PIP meetings have taken place as of March 2023. In Phase 1 the average attendance rate across the six meetings was 76% (5–6 members per meeting), with an increase to 85% recorded for Phase 2 (8–9 members per meeting).

Over 15 SIREN researchers and academic research partners have joined meetings to seek guidance and feedback from PIP members. The timing at which researchers present to the PIP varied, with some approaching the PIP during the design phase and others asking for input about maximising dissemination of their research findings to SIREN participants and the wider public.

### Influence on the SIREN study

Feedback from researchers attending a PIP meeting has been overwhelmingly positive. The PIP has influenced the study design, implementation and evaluation of SIREN and its sub-studies.

### Design phase

While the PIP was not in place prior to the rollout of SIREN, it played an important role in the design phase of SIREN sub-studies. This included a study to understand the clinical impact of COVID-19 on immunosuppressed individuals (the VIBRANT study) [19], a study to understand the impact of winter viruses on healthcare workers, and a study of Group A Streptococcus in healthcare workers.

Involving the PIP at an early stage was found to positively impact the ethics review process through gaining feedback on how to effectively engage with healthcare professionals, including strengthening plans for participant recruitment.

*“We got some fabulous advice right at the start about how to engage with healthcare professionals. The panel’s input probably sped up the whole process of getting ethics approval by about a month. Involving public contributors doesn’t just make a study better, it makes it quicker.” *[20]

- *Researcher 3, VIBRANT Study*

Researchers reflected that PIP feedback strengthened study protocols prior to submission. Drawing on their SIREN experiences, PIP members were able to provide insights into the unintended consequences and practicalities of being a participant, including the impact on their work schedules and acceptability of testing.

*“I attended a PIP meeting to discuss developing a sub-study into Group A Streptococcus carriage. Involving the PIP early in the development of the protocol was invaluable. They helped guide decisions on study design, for example by providing insights into how positive test results would affect different staff members’ working arrangements. They raised specific issues which we had not thought of, for example that taking antibiotics in response to positive results would incur prescription charges for study participants. I shared this feedback with colleagues involved in the project and we incorporated it into our study protocol, strengthening a key document needed for developing the study design.”*

- *Researcher 4, UKHSA SIREN team*

*“The PIP were invaluable when designing the methodology for the SIREN Winter Pressures sub-study. They provided insight into the impact that testing for Influenza and other respiratory pathogens in addition to COVID-19 would have on different staff groups. They asked insightful and probing questions which enabled us to amend the protocol in a way that limited any negative impact whilst still answering our key objectives.”*

- *Researcher 5, UKHSA SIREN team*

### Implementation phase

The PIP directly informed the design of participant engagement activities, and therefore contributed to the success of our cohort retention. Strategies proposed included suggesting plain language summaries to reach a wider audience of participants and summarising key SIREN research papers into an accessible format. SIREN plain language summaries are regularly included in study-wide newsletters and have been well-received by participants.

*“The PIP guided us to reduce the word count but focus in on the important messages, add in eye-catching infographics where possible and get the flow right. Feedback from the PIP reassured us that the final version would be well-received by study participants. We have taken their feedback on board to incorporate into future documents too.”*

- *Researcher 2, SIREN team*

*"I particularly felt our views were listened to when we were discussing the Plain Language summary of SIREN."*

- *Participant 3, SIREN PIP member*

PIP members were invited on an ad hoc basis to contribute to additional SIREN study activities. This included presenting at SIREN participant webinars and taking part in SIREN engagement videos, hosted on a public-facing webpage [21]. The visibility of PIP members helped foster a sense of community amongst SIREN participants and provided reassurance that participants were actively involved in the study and its running.

*“There were spin off opportunities and I have been involved in writing an article and being videoed for the SIREN story.”*

- Participant 4, SIREN PIP member

### Study evaluation

SIREN is an agile study that continued to adapt throughout the evolving COVID-19 pandemic. Examples include the introduction of new research questions as vaccines and variants of concern emerged, and the deployment of Influenza and Respiratory syncytial (RSV) testing to investigate NHS Winter Pressures [22]. SIREN seeks to embed continual evaluation and improvement processes within the study, and the PIP have played a key role in this.

*“The PIP provides an important feedback loop and evaluation role for us. PIP members can offer us insight into how participants may react to changes, and how they actually react once changes have been implemented.”*

- *Researcher 6, UKHSA SIREN team*

PIP feedback highlighted the importance of recognising the contribution of participants to the study. As a direct result, the SIREN team provided sites with tokens of appreciation for participants attending SIREN appointments, including personalised certificates and SIREN study stickers.

### PIP member feedback

**Fig. 3:**
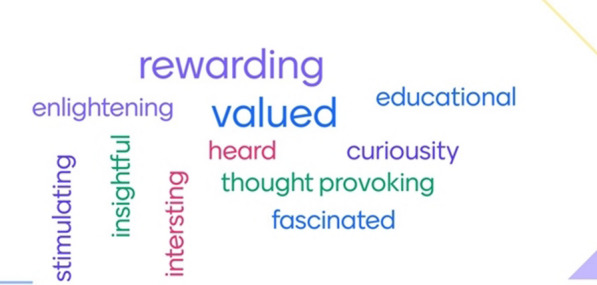
Word cloud based on feedback collected at the final meeting of PIP phase 1. PIP members were asked to describe their involvement with the PIP in three words

Evaluation at the end of Phase 1 found that 100% of PIP members felt that their involvement made a difference to the SIREN study and 100% agreed that the PIP had a positive impact on them as an individual. Top cited words used to describe their experience are included in Fig. 3.

*“I was inspired by discussions and feel privileged to be part of this group.”*

*- Anonymous response from SIREN PIP member, Phase 1 evaluation*

An unintended consequence of taking in part in the PIP was it provided an opportunity for members to reflect on and process their experience of the pandemic.

*“As a PIP member I felt for the first time I could express some of the emotional impact that the pandemic had both personally and on my speciality as a respiratory physician.”*

Participant 5, SIREN PIP member

Participants attributed their positive experience to seeing the tangible impact from their involvement. For example, through researchers returning to a second meeting and providing an update about their work and how feedback from the PIP was incorporated.

*“Even more powerful was the ‘you said, we did’ element to meetings in which the same researchers would return and tell us how they had utilised our feedback and taken on board the issues we had raised to improve the study aims and plans. This made me feel like we had really made a difference and had not only been listen to but been heard.”*

- *Participant 4, SIREN PIP member*

## Discussion

SIREN is an ambitious UK-wide prospective cohort study that was established at pace in 2020 to evaluate the immune response to SARS-CoV-2 amongst healthcare workers. Our cohort of healthcare workers are crucial to the success of the study and SIREN recognises the importance of actively involving participants in our research. People who the research is for an about offer a unique perspective, and working in partnership with them can positively influence study design and implementation, resulting in higher quality research.

The COVID-19 pandemic resulted in the rapid funding and rollout of research studies. However, the rate of involvement from patients, carers, service users or other members of the public in research studies submitted for approval dropped sharply for COVID-19 research, while other aspects of good practice remained in place [23]. The Health Research Authority (HRA) reported that prior to the pandemic 80% of research applications typically documented the involvement of patients and the public; however, this reduced to just 22% for urgent COVID-19 studies submitted in March–April 2020 [14].

Despite the rapid timescales for delivery, within SIREN we recognised the value of actively involving a panel of participants in the study, particularly given its scale and the role SIREN has played in informing the national pandemic response. We acknowledged that the pandemic impacted all levels of society and had an unprecedented impact on individuals’ everyday life, while also affecting population health and increasing the burden on health and care resources [24]. As such, it would be remiss not to provide the subjects of research studies with a voice and an opportunity to contribute actively through participant involvement work. This is particularly true for research studies involving healthcare workers who were at the frontline of the pandemic response and were disproportionately affected, carrying a heavy disease burden [25]. Our experience in SIREN demonstrated how studies established during a pandemic may evolve with the emergence of novel research questions and that participant involvement is invaluable in ensuring the research remains relevant and feasible.

This paper aimed to provide a narrative account of our approach to participant involvement in a large, multicentre pandemic response cohort study, demonstrating the influence and value of the SIREN PIP. Exploring its influence on PIP members and the study itself has identified key areas of consideration for establishing participant involvement in a pandemic response study, contributing to future pandemic preparedness (Table 1).

**Table 1:** Thematic grouping of learning for future pandemic preparedness research studies

Dedicated resources	• Sufficient funding to provide dedicated staff resource, external expertise if required, and to follow good practice guidelines for recognising the contribution of participants
Recruiting the right panel	• Being clear in who your study needs to hear from to help establish clear criteria for recruiting panel members
Understanding motivations for participant involvement	• Research focusing on a pandemic response can invoke altruism and encourage active participation in research
Flexible options for participant involvement	• Adapting opportunities for involvement to suit participants, including considering practicalities such as timing and length of meetings
Early involvement from participants	• Participant involvement in study design can strengthen research methodology and make it more acceptable for uptake by participants

SIREN collaborated with the BSI, who have a strong track record in delivering participant engagement and involvement [20]. This ensured we recruited the right panel of participants which has enabled our research to remain relevant to policy makers, the public and scientific community.

The SIREN PIP was open to applications from the whole cohort, which includes staff working at different levels of seniority, pay grades and contractual arrangements. Offering an honorarium encouraged participation from across staff groups and helped make participants feel valued.

*“Being offered an honorarium payment was more than just the money. It made me feel that my time was valued, and I know that some of the members donated their payment to charities.”*

- Participant 4, SIREN PIP member

Learning from Phase 1 generated criteria for Phase 2 recruitment. Referencing these criteria in study communications, particularly with respect to encouraging applications from people from ethnic minority groups, people with disabilities and people with gender diverse identities, resulted in a panel that was better able to reflect the variety of participant experiences.

*"The group was made up of a range of people, covering healthcare sectors, such as medicine, nursing, research and academics. It was specifically formed to represent the four countries, but everyone’s opinions were equally heard."*

- Participant 4, SIREN PIP member

This considered approach to recruitment resulted in PIP members who did not feel constrained by background or hierarchy.

*"The NHS is traditionally a very hierarchical institution. One can easily be influenced or intimidated by someone perceived to be in a position of greater influence or experience. It quickly felt like we were all on a level playing field. All views counted."*

- Participant 1, SIREN PIP member

A common theme of altruism and desire to contribute to the pandemic response emerged from PIP member feedback, and from those who expressed an interest in joining the panel. We have reflected that appealing to this goodwill and desire to help could be an important feature of recruitment strategies in future pandemic response studies.

*"For me the overriding feeling was one of improving things for future generations and it became clear that the studies that we heard about would do this…I am proud to have been involved in this small way to improve things for my children and my children’s children. In some way some positive has come out of this dreadful pandemic and my involvement in the PIP has been quite cathartic as it has righted some of the terrible wrongs that occurred for so many people in the pandemic."*

- *Participant 4, SIREN PIP member*

Consideration needs to be given to practical elements of PIP member involvement, to ensure there are flexible options for contributing. Holding panel meetings virtually encouraged sustained engagement from participants who could join from any location. SIREN PIP members joined meetings from the office, from home and even from holiday destinations – demonstrating the strength of engagement and dedication from participants.

SIREN took a practical approach to PIP meeting attendance. It was understood that PIP members may not be able to attend all meetings. We provided alternative options for PIP member involvement, including accepting feedback pre or post meeting via email, and encouraging participation in wider study activities such as webinars and videos.

Rapid development and rollout of studies is common in pandemic settings. This can make it challenging to allocate time for the recruitment of appropriate individuals for participant involvement activities [20], particularly in the early stages of study design and planning. Future efforts should include establishing a national PIP group to contribute to the early stages of study design in future pandemics.

### Limitations

The SIREN cohort is predominately white and female with a high percentage of nurses compared to other professional groups. While this is an accurate reflection of the NHS working population, we understand this does not reflect the UK wide population. In addition, SIREN was established at scale and pace in response to an emerging and evolving pandemic. As a result, the PIP was established at a later stage in the study once set-up was complete, and all participants had been recruited. This meant that the study was unable to seek participant guidance and feedback in the early phases of study design.

## Conclusions

The SIREN PIP have demonstrated the value of actively involving people who research is for and about. We have demonstrated its role providing a feedback mechanism for research at key stages of the study and sub-studies, including during the design, implementation and evaluation stages of research. Participant involvement should also benefit individuals taking part and this has been achieved during the SIREN study. This paper makes the case for participant involvement in future pandemic research studies. Future work should include improved training for researchers in establishing and running participant involvement panels, and we would support the development of a national PIP forum as part of future pandemic research preparedness.

### Supplementary Information

**Additional file 1. **GRIPP2 PPI Long Form reporting checklist.

## Data Availability

Not applicable.

## Abbreviations

BSI: British Society for Immunology
NIHR: National Institute for Health and Care Research
PIP: Participant involvement panel
PPI: Patient and Participant Involvement
RSV: Respiratory Syncytial Virus
SIREN: SARS-Cov2 Immunity & Reinfection Evaluation
UKHSA: UK Health Security Agency
